# High Pyrethroid Resistance in *Triatoma infestans* Is Strongly Associated with *kdr* Variants: A Multiplex High-Resolution Melting Tool for Vector Surveillance

**DOI:** 10.3390/insects17090969

**Published:** 2026-09-20

**Authors:** Ivana Sierra, Lucila Traverso, Anneris Gomez, Patricia Alejandra Lobbia, Gastón Mougabure-Cueto, Gonzalo Domínguez, María Soledad Santini, Sheila Ons

**Affiliations:** 1 Laboratorio de Neurobiología de Insectos, Centro de Endocrinología Experimental y Aplicada, Facultad de Ciencias Médicas—Facultad de Ciencias Exactas, Universidad Nacional de La Plata—Consejo Nacional de Investigaciones Científicas y Técnicas (CONICET), Bvd. 120. Num 1459, La Plata 1900, Argentina; sierra.ivana@gmail.com (I.S.); lucilamtraverso@gmail.com (L.T.); ghdominguez@med.unlp.edu.ar (G.D.); 2 Instituto Nacional de Parasitología “Dr. Mario Fatala Chaben” (INP)-Administracion Nacional de Laboratorios e Institutos de Salud (ANLIS)-Malbran, Consejo Nacional de Investigaciones Científicas y Técnicas (CONICET), Av. Paseo Colón 568, Ciudad Autónoma de Buenos Aires 1063, Argentina; anneris.gomez89@gmail.com (A.G.); mariasoledadsantini@gmail.com (M.S.S.); 3 Cátedra Morfología Animal, Facultad de Ciencias Exactas, Físicas y Naturales Universidad Nacional de Córdoba, Av. Vélez Sarsfield 299, Córdoba 5000, Argentina; patalobbia@gmail.com; 4 Instituto de Investigaciones Biológicas y Tecnológicas (IIBYT-CONICET), Av. Vélez Sarsfield 1611, Córdoba 5000, Argentina; 5 Laboratorio de Fisiología de Insectos, Departamento Biodiversidad y Biología Experimental (DBBE), Facultad de Ciencias Exactas y Naturales, Instituto de Biodiversidad y Biología Experimental y Aplicada (IBBEA, UBA-CONICET), Universidad de Buenos Aires, Intendente Güiraldes 2160, Ciudad Autónoma de Buenos Aires 1428, Argentina; gmougabure@gmail.com

**Keywords:** voltage-gated sodium channel, insecticide, vector management, L1014F, L925I

## Abstract

*Triatoma infestans* is the main vector of *Trypanosoma cruzi* in the Southern Cone, where vectorial transmission of Chagas is still occurring despite control campaigns with pyrethroid insecticides. High pyrethroid resistance was detected in *T. infestans* populations from Argentina and Bolivia. This raises the urgent need for including strategies for monitoring and management of insecticide resistance as an essential component of control campaigns. Voltage-gated sodium channels are the target site of pyrethroids; non-silent single-nucleotide polymorphisms in specific sites of the gene encoding this channel, called kdr mutations, are associated with resistance to pyrethroids in many insect species, including *T. infestans*. The early detection of kdr in a population allows taking control decisions timely. In this work, we present the development of a high-throughput assay based on multiplex high-resolution melting for genotyping kdr variants in this species. We characterized *T. infestans* populations according to their toxicological response to deltamethrin and efficiently used this assay for genotyping. Our results indicate that high resistance to pyrethroids is closely related to the presence of kdr variants in *T. infestans,* and they provide a high-throughput method for vector surveillance and resistance management.

## Simple Summary

Chagas disease affects millions of people worldwide; it is spread by blood-feeding insects known as kissing bugs, which are endemic in Latin America. In the Southern Cone, the main vector is *Triatoma infestans*, which lives inside houses and is controlled by spraying dwellings with pyrethroid insecticides. In some regions of Argentina and Bolivia, these insects have become highly resistant to those insecticides, so spraying no longer kills them and transmission continues. In this species, resistance has been closely associated with mutations in the gene that encodes the voltage-gated sodium channel, which is the target site of pyrethroids. Detecting those changes early, before they spread through a whole population, would let health authorities adjust their strategy in time. Here, we developed a cost-effective assay that identifies the two known resistance-causing changes at once, in a single reaction. We examined insects from several Argentine and Bolivian regions and found that these genetic changes appeared only in populations highly resistant to the insecticide, never in susceptible ones. The new test is quicker and cheaper than existing methods based on enzymatic digestion or allele-specific PCR, despite the requirement of more expensive equipment. It could be incorporated into control programs to monitor resistance routinely.

## 1. Introduction

Chagas disease was recognized as a neglected tropical disease (NTD) in 2005 by the World Health Organization (WHO) and was subsequently included in its overall plans and roadmaps for neglected tropical diseases [1]. It is caused by the protozoan parasite *Trypanosoma cruzi*, which is transmitted to humans through the feces of triatomine insects (kissing bugs) during feeding on host blood. The disease affects around 8 million people over the world, being endemic in tropical areas of Central and South America; global warming and population movements spread Chagas to non-endemic areas [2]. Thirty to 40% of people living with Chagas develop cardiac and/or digestive disease, which can cause disability and death. Despite efforts in control campaigns using pyrethroid insecticides, vectorial transmission of *T. cruzi* could not be stopped in wide areas of the Southern Cone, where *Triatoma infestans* acts as a domiciliary vector. Insecticide resistance and domiciliation of *T. infestans* in both urban and rural dwellings are important challenges for the prevention of *T. cruzi* transmission [3,4].

Spraying of infested dwellings with pyrethroid insecticides has been the method of choice for triatomine control for the last 30 years, given the favorable toxicological properties of these insecticides [4]. Since 2005, high pyrethroid resistance has been reported in the Bolivian–Argentinean border and in the Chaco province in Argentina, regions where both infestations of dwellings with *T. infestans* and *T. cruzi* transmission occur [5,6]. In Argentina, alternatives to pyrethroids have been banned since 2017 for domi-sanitary use [7], given toxicological and operational considerations; this makes pyrethroid resistance a relevant problem for public health, especially in our country. Integrated vector management strategies are recommended by the WHO for efficient and sustainable control of arthropod vectors [8]. Among these strategies, the early monitoring of resistance is essential for a cost-effective and integrated vector management [8]. The diagnosis of single-nucleotide polymorphisms associated with resistance allows early detection, given that the detection of an individual carrying the mutation, before the mutation is fixed in the population, can be achieved.

**Figure 1:**
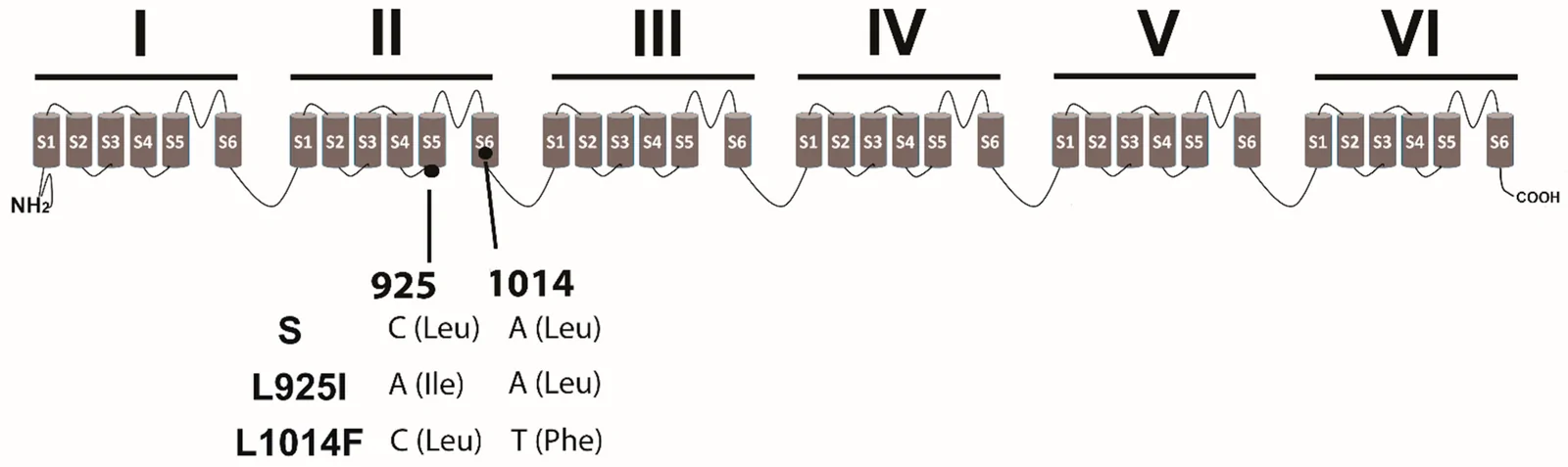
*
Triatoma infestans* voltage-gated sodium channel scheme. Positions of the variants 925 and 1014 are indicated. For each variant, the corresponding nucleotide and encoded amino acid (between brackets) are indicated. The channel is represented with domains I–IV, each with the six transmembrane segments (S1–S6).

Pyrethroid insecticides act through the voltage-gated sodium channel (VGSC), present in the membranes of excitable cells. Genetic variants in the gene encoding this channel have been associated with high levels of pyrethroid resistance in insects [9,10]. These mutations are called knockdown resistance (kdr) variants, given that they confer low sensitivity to pyrethroids and consequent resistance to knockdown [9,10]. Most kdr mutations in insects were found in domain II of this large membrane protein, particularly in the region comprised between transmembrane segments IV and VI (IIS4-IIS6 region). In our previous work, we described two kdr mutations located in domain II of the gene (Figure 1): Leu to Ile in position 925 (L925I), present in resistant populations from the Argentinean Chaco, and Leu to Phe in position 1014 (L1014F), characteristic of resistant populations in the Bolivian–Argentinean border [11,12,13]. Even though other contributive mechanisms could exist [14,15,16,17], kdr is the main insecticide-conferring resistance mechanism known for triatomines reported to date.

Sensitive detection of pyrethroid resistance is a prerequisite for vector management. For this, the genotyping of kdr variants is a valuable tool, faster and more sensitive than toxicological determinations [18]. In our previous work, we have developed two methods for the molecular diagnosis of L925I and L1014F variants; these methods are based on differential digestion by restriction endonucleases (REA) or allele-specific PCR (PASA), respectively [11,12,13]. Both methods are robust and can be carried out in laboratories with basic equipment. However, real-time PCR-based methods, such as multiplex high-resolution melting (mHRM), could improve the throughput of genotyping by resolving both mutations in a single PCR reaction, followed by the analysis of melting curves (MCs) [19].

HRM is a high-throughput genotyping technique, based on the interpretation of the MC produced during the denaturation step, after PCR amplification with a double-strand DNA dye [20]. Given the high sensitivity of the technique, single mutations will cause distinguishable melting temperatures (MTs) and/or different shapes in the MC, which are more evident whenever the substitution is a guanine/cytosine by adenine/thymidine. However, a possible drawback of the HRM method is that MC interpretation could be challenging and time-consuming, mainly when the substitution occurs between two purinic or two pyrimidinic bases, given the smaller shift in the MT of the amplicons.

In this work, we present the development of an efficient mHRM-based assay to genotype kdr mutations in *vgsc* from *T. infestans.* We compared HRM with previous methods regarding their performance and cost-efficiency. Finally, we determined the pyrethroid resistance status of different natural populations from Argentina and tested the presence of kdr variants in these populations by mHRM. Our results confirm that kdr mutations strongly correlate with a high-resistant phenotype in the species and indicate that mHRM is a cost-effective method for genotyping these mutations in *T. infestans*. We conclude that kdr genotyping by mHRM could be incorporated for resistance monitoring and management of *T. cruzi* vectors in the Southern Cone.

## 2. Materials and Methods

### 2.1. Insects

**Figure 2:**
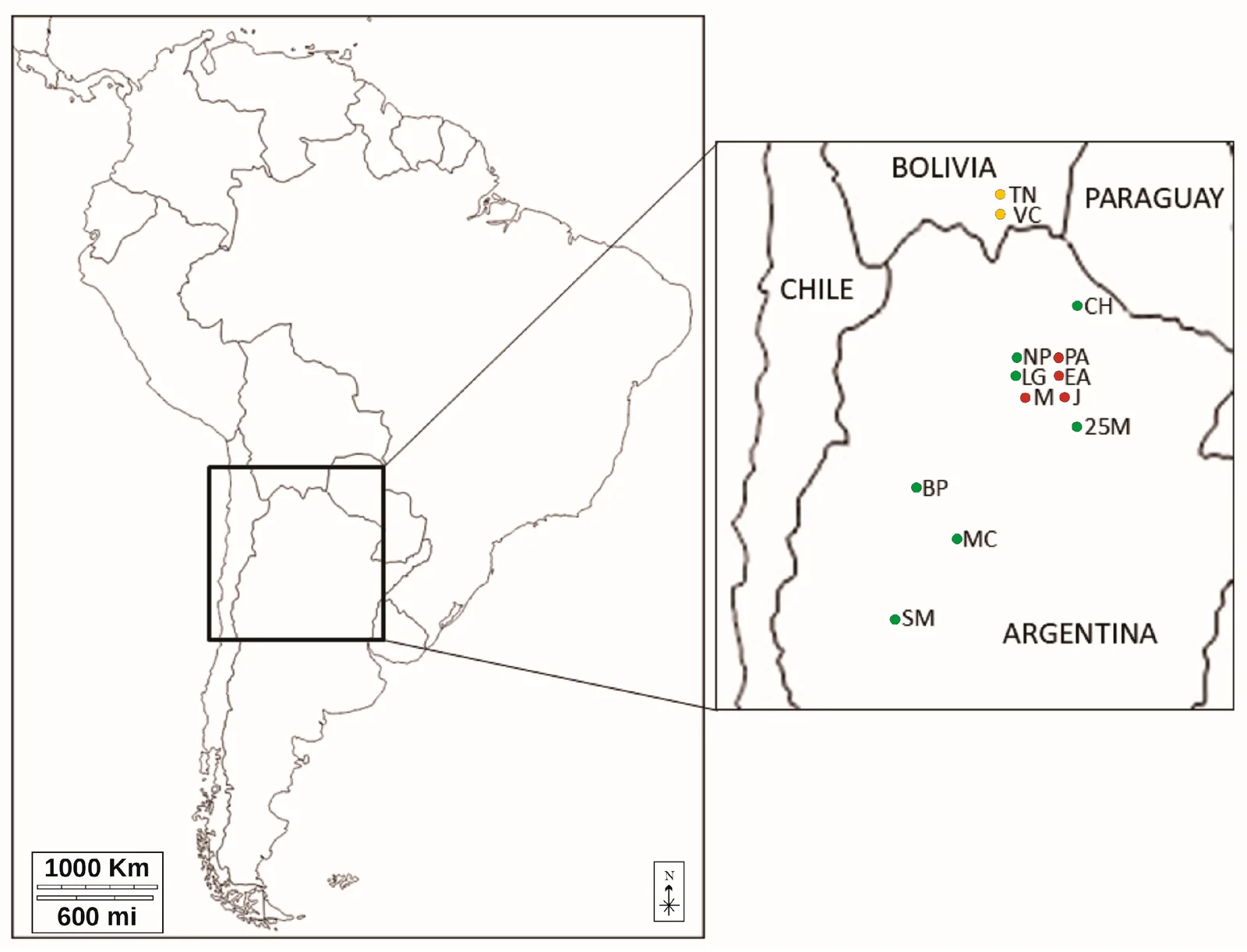
Geographic distribution of the populations that originated the strains assayed in this study. Green circles indicate susceptibility; red circles indicate highly resistant L925I^kdr^; yellow circles indicate highly resistant L1014F^kdr^. Argentinean populations: 25 M; 25 de Mayo; BP: Balde de Punta; CH: Chiriguanos; EA: El Asustado; J: El Juramento; M: El Malá; LG: La Gerónima; MC: Minas; SM: San Martín; NP: Nueva Pompeya; PA: Pampa Argentina. Bolivian populations: TN: Tierras Nuevas; VC: Villa del Carmen.

*T. infestans* were collected from Argentinean localities (Figure 2) as previously described [11]. For the populations from El Juramento, La Gerónima, El Malá, and the Bolivian populations from Tierras Nuevas and Villa del Carmen, DNA previously obtained in that study was used; the remaining populations analyzed were genotyped here for the first time. The insects collected in the field were raised at the former Centro de Referencia de Vectores (Córdoba, Argentina) under controlled conditions (26 ± 1 ° C, 60 ± 10% RH, and 12:12 LD). For colony maintenance, all the insects were fed on chickens every 15 days. The corresponding protocol for chicken care has been officially submitted to the Institutional Animal Care and Use Committee (CICUAL-FCE-UNLP) and is under registration number 005-00-26. The housing, care, feeding, and handling of the chickens adhered to Resolution 1047/2005 (CONICET, https://www.conicet.gov.ar/wp-content/uploads/OCR-RD-20050701-1047.pdf (accesed on 17 September 2026). This resolution outlines the national ethical framework for biomedical research involving laboratory, farm, and wild animals, and is consistent with international standard procedures. Biosafety measures were in accordance with CONICET Resolution 1619/2008, which aligns with the World Health Organization Laboratory Biosafety Manual (https://www.who.int/publications/i/item/9789240011311, accesed on 17 September 2026).

### 2.2. Toxicological Determinations

The insects collected in the field were raised until the F1 was obtained. The toxicological status of each population was determined by topical application of 0.01 mg/mL of deltamethrin in acetone, which is the discriminating dose (DD) for *T. infestans*. DD was applied by the topical bioassay according to the WHO protocol [21]. Briefly, a volume of 0.2 μL (2 ng) of technical grade deltamethrin (95.5 %; Merck, Autónoma de Buenos Aires, Argentina) in analytical grade acetone (Merck, Argentina) was applied on the dorsal abdomen of first-instar nymphs (5–7 days old, mean weight 1.3 ± 0.2 mg) starved since hatching. The application was performed with a 10 μL Hamilton syringe (Hamilton, Reno, NV, USA) provided with a repeating dispenser. Ten insects were used for each replicate, and each sample was replicated at least three times. Control groups received pure acetone. After exposure, insects were kept under laboratory conditions for 24 h, when mortality was evaluated. The criterion for mortality was the inability to walk from the center to the border of a circular 11 cm diameter filter paper. Whenever 100% of the individuals died at DD, the population was considered susceptible. For resistant populations, at least 4 doses were applied within a range that provoked mortality from 10% to 90%. Dose–mortality data were subjected to probit analysis [22] to estimate the lethal dose required to kill 50% of treated individuals (LD50) using POLO PLUS Software (LeOra 2007). Mortality data were corrected using Abbot’s formula implemented in the POLO PLUS package. Resistance ratios (RR50s) and 95% confidence limits (CIs) were calculated as described [23]. The differences between susceptibilities of two strains were evaluated through the Lethal Dose Ratio (LDR) [23]. The LDR and its 95% CIs compare dose–response curve pairs and are based on estimates of each line (i.e., slope and its variance, intercept and its variance, and the slope–intercept covariance) and a coefficient according to the level of response in which the lines are compared (50% mortality level). Thus, the LDR estimates the susceptibility ratio between the strains compared. The LDR is significantly different from 1 when the 95% CIs did not include the number 1 (*p* < 0.05). In the context of evaluation of resistance, the LDR is named the resistance ratio (RR). A given population was considered resistant when RR50s significant differs from 1. Like in a previous report [24], RR50 < 10 was classified as low resistance, and RR50 > 100 was considered high resistance; RR > 10 > 100 (middle resistance) was not observed in the populations studied here. Even though these cutoffs for classifying levels of resistance were previously implemented [24], they should be considered arbitrary by definition. However, the populations studied here are clearly separated according to their toxicological response in sensitive, low, and high resistance.

### 2.3. DNA Extraction

For the extraction of genomic DNA, three legs and one antenna were dissected from each insect; in this way, we kept backup material from each individual and avoided the use of complete heads and other structures which could carry pigments potentially inhibitors of qPCR. A total of 10 insects from each population were pooled for genomic DNA extraction with the commercial Quick Genomic DNA Extraction Kit (Dongsheng Biotech, Guangzhou, China) following the manufacturer’s instructions.

### 2.4. mHRM Assay Development

Primer3 v.0.4.0 software (https://primer3.ut.ee/, accessed on 17 September 2026) was used for the primer design flanking the positions named 925 and 1014 of the *vgsc* gene from *T. infestans* (see primer sequences in Table 1). Amplicons’ MTs were predicted with uMELT online software (https://www.dna-utah.org/umelt/quartz/, accessed on 17 September 2026). GC tails were added to the forward and reverse primers of the 1014 amplicon to differentiate at least 2 °C MT both amplicons.

Plasmidic DNA was used for setting the mHRM reaction. Standard templates were prepared based on the strategy described previously [11]. Briefly, genomic DNA fragments encompassing the L1014F or L925I resistance alleles were PCR-amplified from pools of 10 insects with known genotypes from Villa del Carmen (L1014F) and El Juramento (L925I) populations. The PCR products were cloned into pGEM-T (Promega, Fitchburg, WI, USA) plasmids, and the resulting plasmids were used to generate standards with defined resistant: susceptible molar ratios for each position. The standard DNA templates were mixed at the following molar ratios: 0:10, 1:9, 2:8, 3:7, 4:6, 5:5, 6:4, 7:3, 8:2, 9:1, and 10:0 (resistant allele:susceptible allele at each mutation site). The identity of the cloned inserts was confirmed by Sanger sequencing (Macrogen, Seoul, Republic of Korea).

Both the RT-PCR and mHRM reaction steps were performed on the AriaMx Real-time PCR instrument (Agilent, Santa Clara, CA, USA). PCR was performed in 12 μL containing 6 μL Eva green master mix (Agilent, CA, USA) and either plasmidic (10 ng) or genomic DNA (40–100 ng). The primer concentrations were optimized individually for the specific amplicons, and later in the mHRM, in order to obtain both amplicons at a similar concentration. The following primer combinations were assayed (expressed as concentration of primer pair 925/concentration of primer pair 1014): (a) 150 nM/300 nM; (b) 300 nM/150 nM; and (c) 200 nM/200 nM. For further mHRM assays, the first alternative was used. The PCR amplification protocol began with a denaturation step at 95 °C for 3 min, followed by 40 cycles of 5 s denaturation at 95 °C and 30 s annealing and extension at 62 °C. After amplification, the reaction tubes were cooled to 55 °C and then warmed from 55 °C to 95 °C at the rate of 0.2 °C/second. The MCs were analyzed with AriaMx 1.5 software (Agilent, CA, USA). Plasmids carrying inserts of known genotypes were used as standard reference in all the reactions. The standard DNA templates of known genotypes inserted in plasmids were pooled in known proportions of “kdr” and “susceptible” genotypes in each mutation position (925 or 1014). Samples of 100%, 90%, 80%, 70%, 60%, 50%, 40%, 30%, 20%, 10%, and 0% of kdr mutation were obtained for each one of these sites.

### 2.5. Statistical Analysis

For melting temperature comparisons, the susceptible groups comprised 9 independent samples in position 925 and 11 in position 1014. The resistant groups comprised 4 independent samples in position 925 and 2 independent samples in position 1014. Each population-level DNA pool was analyzed in technical mHRM replicates. Technical replicates were treated as independent observations in the *t*-test, given that within-population variability was negligible compared to the genotype-associated shift in melting temperature. Differences in MT between amplicons carrying different variants in genomic DNA were analyzed using a *t*-test, with Welch’s correction when variances were not homogeneous according to the Shapiro–Wilk test, with Prism 8.0 software (GraphPad Software).

## 3. Results

### 3.1. Setting mHRM Reaction

**Figure 3:**
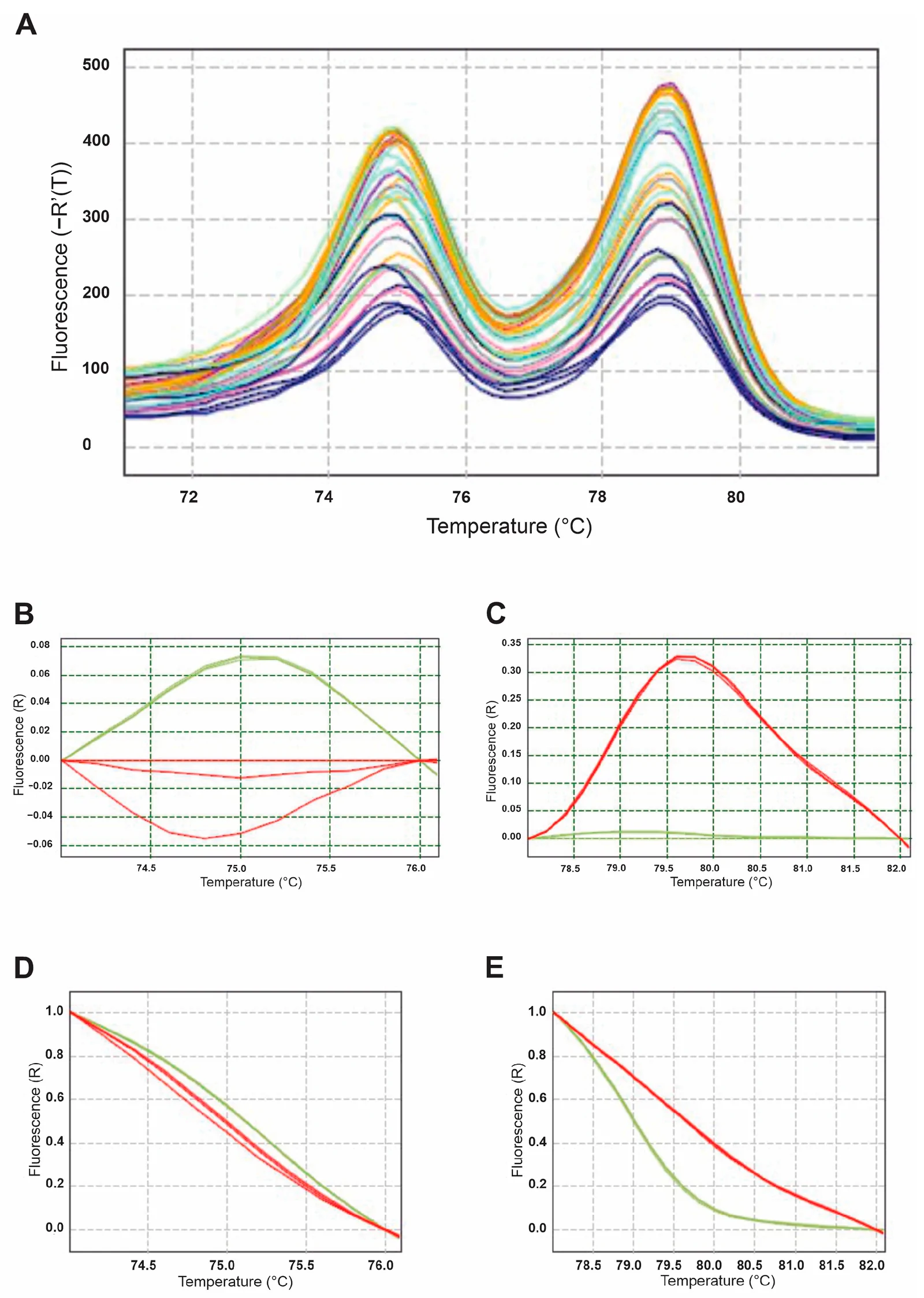
Results of genotyping 925 and 1014 positions in the *T. infestans vgsc* gene with mHRM. Detection of kdr variants by melt curve analysis of standard samples. (**A**) Raw results of mHRM. Peaks on the left and on the right belong, respectively, to 925 and 1014 amplicons. (**B**) Difference melting plots for amplicons with variation in the 925 position (green: L925 variant; red: L925I^kdr^ variant). (**C**) Difference melting plots for amplicons with variation in the 1014 position (green: L1014 variant; red: L1014F^kdr^ variant). (**D**) Melting curve for amplicons with variation in the 925 position (green: L925 variant; red: L925I^kdr^ variant). (**E**) Melting curve for amplicons with variation in the 1014 position (green: L1014 variant; red: L1014F^kdr^ variant).

Plasmids carrying an insert of a known genotype were used as a standard for setting the mHRM reaction. Both the 925 and 1014 positions were amplified in duplex PCR, with two primer pairs in a single reaction tube. By the addition of GC tails in the 5′ regions of 1014 forward and reverse primers (Table 1), a difference in MT of nearly 3 °C between 925 (MT 75.11+/−0.03 °C) and 1014 (MT 78.87+/−0.02 °C) amplicons was achieved (Figure 3A). This difference allowed the individual analysis of both polymorphic sites using two primer pairs in a single reaction.

As expected for a substitution of a cytosine (in the wild-type) by an adenine (in the kdr variant), melting curves of the amplicon in the 925 position showed a shift of −0.4 °C when the kdr genotype L925I^kdr^ is present. MT was around 74.8 °C for samples carrying a cytosine (sensitive-like) and 74.4 °C for the amplicons carrying an adenine (L925I^kdr^-like). The shift in MT was smaller in the 1014 position than in the 925 position when the substitution associated with L1014F^kdr^ was present, given that the mutation conferring resistance is an adenine for a thymidine. This would indicate that the genotype in this position could not be easily distinguished by MT shift. The shape of the melting curve was different depending on the genotype of the amplicon in both the 925 and 1014 positions, even though this difference in shape is better distinguishable in the latter (Figure 3B–E). Hence, despite the low or undetectable change in MT, L1014F^kdr^ substitution could be detected by analyzing the shape of the melting curve.

**Figure 4:**
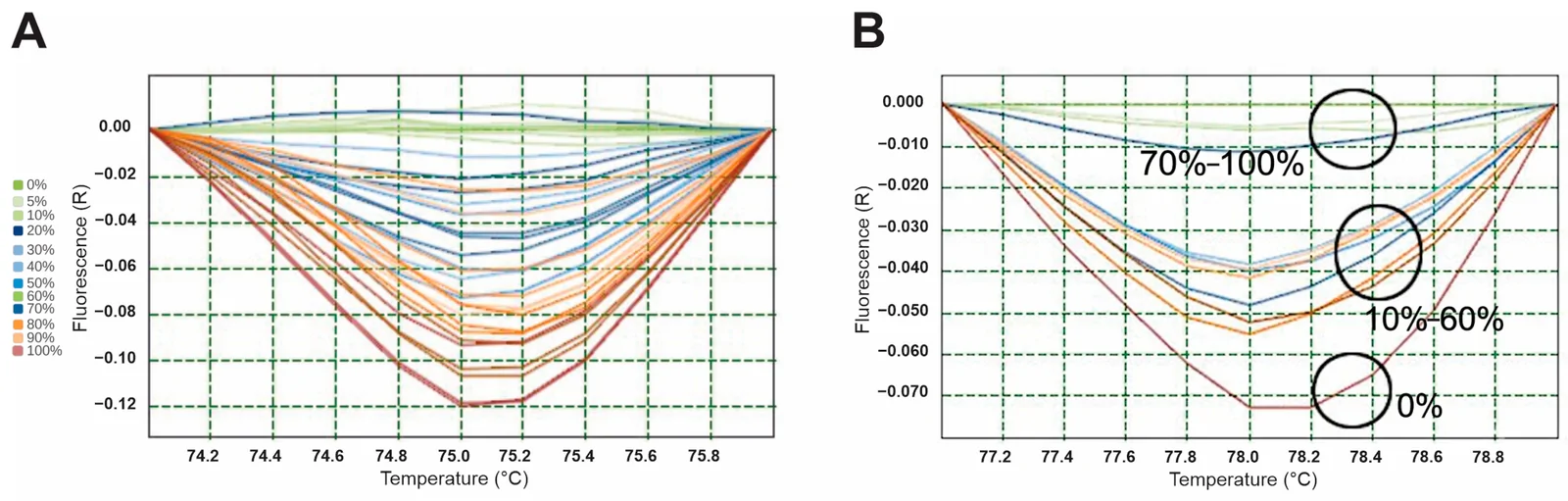
Difference in melting temperature plots obtained from DNA mixtures containing increasing proportions (0–100%) of the resistant genotype. (**A**) DNA mixtures containing increasing proportions of the L925I^kdr^ variant. (**B**) DNA mixtures containing increasing proportions of the L1014F^kdr^ variant, showing limited discrimination of intermediate genotype proportions.

We studied the sensitivity of the assay for detecting the resistant mutations in a sample with complex allele composition. For this, a set of standard DNA template mixtures of known genotype was adjusted to contain an increasing frequency of one resistant mutation: 0; 10; 20; 30; 40; 50; 60; 70; 80; 90; and 100%. This strategy was repeated for both mutations. For the 925 position, increasing the percentage of the L925I genotype provoked a gradual shift to lower MT (Figure 4A). For this position, a linear correlation (R^2^ = 0.97) was observed between MTs and genotype proportions of the kdr mutation in the sample. This would potentially allow for the estimation of the percentage proportion of mutant DNA in pooled samples by comparing with a standard curve. In the case of the kdr mutation in the 1014 position, the proportion between melting temperature and percentage of DNA carrying the mutation does not fit a linear regression (Figure 4B). In any case, to confirm frequencies of both L925I^kdr^ and L1014F^kdr^ mutations in mixed samples, individuals should be analyzed separately.

### 3.2. Toxicology and Genotyping of Wild Populations

Six natural *T. infestans* populations from five Argentinean provinces were evaluated to determine their resistance status by a toxicological assay (Figure 2; Table 2). The localities studied are located in La Rioja (San Martín), Catamarca (Capayán), Córdoba (Minas), Formosa (Chiriguanos), and Chaco (Quitilipi and Nueva Pompeya); all of them were classified as sensitive according to their response to a DD of deltamethrin.

As suggested by a previous report [11], we hypothesized that high resistance in *T. infestans* populations correlates with either the L925I^kdr^ or L1014F^kdr^ variants. To test this hypothesis, individuals obtained from sensitive, low-resistant, or high-resistant populations were evaluated by mHRM (Table 2). In addition to the samples from populations evaluated here for the first time at the toxicological level, we included samples previously classified as sensitive, low, or highly resistant (Table 2; Figure 2).

**Figure 5:**
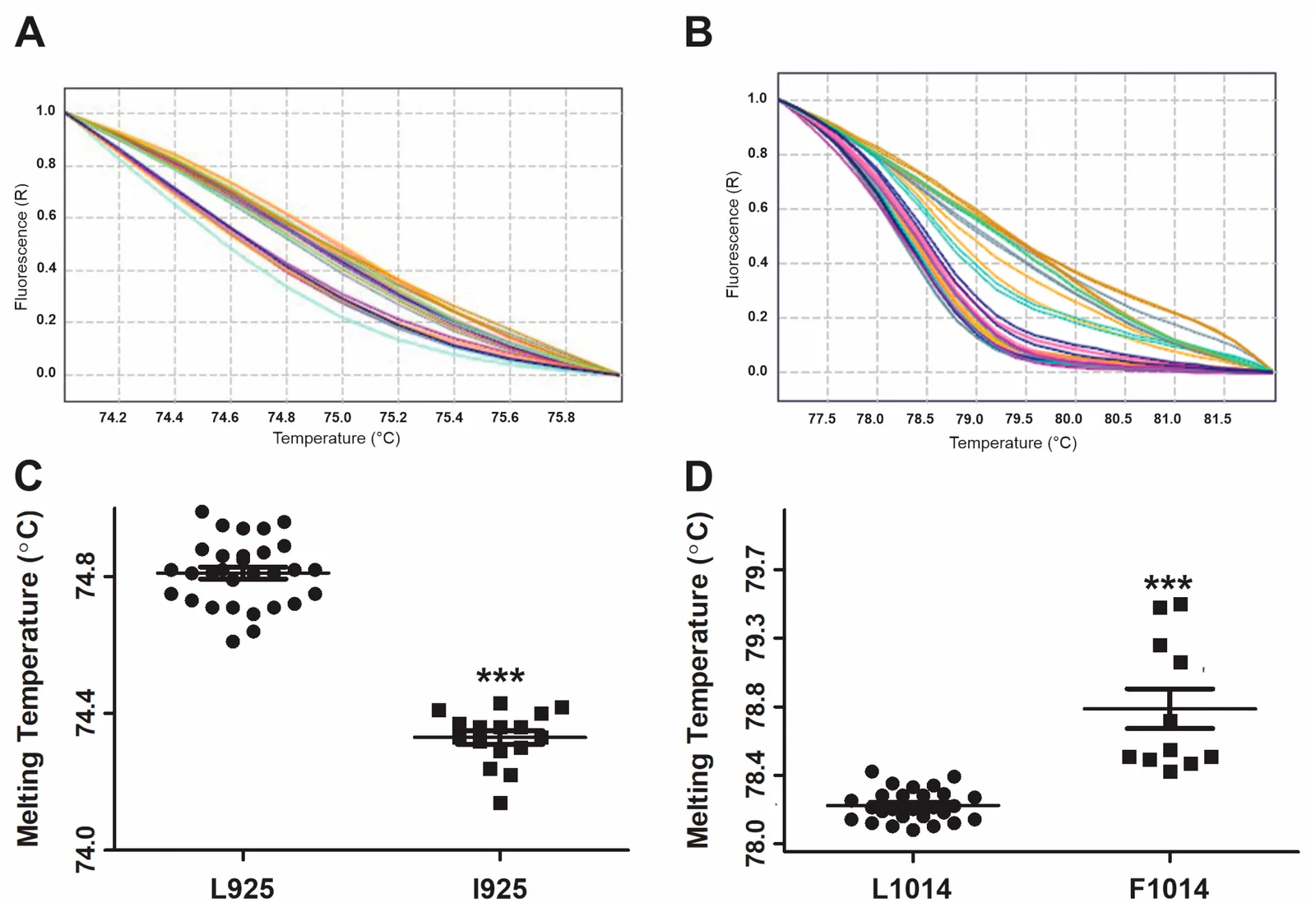
Results of genotyping at positions 925 and 1014 in the *T. infestans vgsc* gene. mHRMs were performed using genomic DNA extracted from populations presenting different toxicological phenotypes. Each dot represents a single technical replicate. (**A**) Melting curves for amplicons in the 925 position. (**B**) Melting curve for amplicons in the 1014 position. (**C**) Melting temperatures of samples classified as susceptibles (S) or L925I^kdr^. *** = *p* < 0.0001 (*t*-test). (**D**) Melting temperatures of samples classified as susceptibles (S) or L1014F^kdr^. *** = *p* < 0.0001 (*t*-test with Welch’s correction).

In the pooled samples, the characteristic changes in the shape of the melting curve for both the 925 and 1014 positions were observed (Figure 5A,B). Wild-type samples were distinguished from samples carrying the L925I^kdr^ variant by the expected significant shift in MT; 74.81 (+/−0.02) °C for S and 74.33 (+/−0.02) °C for L925I^kdr^ (*t*-test, *p* < 0.0001, N = 16–30) (Figure 5C). For the 1014 position, the shift in MT (78.27 (+/−0.02) °C for samples classified as S and 78.84 (+/−0.11 °C) for samples classified as L1014F^kdr^ also reached statistical significance (*t*-test, *p* < 0.001, N = 27–11) (Figure 5D).

Interestingly, we found that all populations classified as susceptible or low-resistant showed the susceptible mHRM profile in both the 925 and 1014 positions. Conversely, the populations classified as highly resistant from Argentina showed the resistant mHRM profile in position 925, and those samples from the Bolivian highly resistant populations showed the resistant mHRM profile in position 1014 (Table 2).

## 4. Discussion

Insecticide resistance spreading is a challenge for the vector control programs; for this reason, strategies to delay resistance must be implemented in rational control campaigns. Monitoring of resistance-associated mutations in the field is a requisite for the implementation of management strategies that prolong the utility of insecticides. This information is an important resource for successful decision-making on control strategies.

*T. infestans* demonstrated a potential to develop extremely high levels of resistance to pyrethroids [3,6], which is recognized as one of the main challenges for stopping the vectorial transmission of *T. cruzi* in the Southern Cone [28]. The results presented here (Table 2) are in agreement with previous reports [11,12,13], indicating that this extremely resistant phenotype is strongly associated with kdr mutations in the *vgsc* gene. In that sense, the kdr variants could be considered a promising molecular marker for surveillance of high pyrethroid resistance, a relevant information for decision-making in vector control campaigns. The genotyping of kdr could be rapid, straightforward, and sensitive when compared with toxicological determinations. For this, the development of high-throughput genotyping methods will be necessary.

In our previous work, we provided two molecular genotyping methods based on PASA and REA to genotype L1014F^kdr^ and L925I^kdr^ in *T. infestans*, respectively [11,12,13]. Here, we present a new method based on mHRM that augments the throughput in those laboratories with access to a well-calibrated real-time PCR system. A comparison of PASA + REA and mHRM for the genotyping of both kdr mutations is provided in Table 3. Both methods are efficient and reliable; the main advantage of mHRM is its higher throughput and lower cost. This is due to the characteristic “one-step” closed-tube method that allows the genotyping of both kdr mutations in a single reaction. When high or fixed frequencies in the 925 and 1014 positions in VGSC in *T. infestans* are suspected, pooled analysis (i.e., up to 10 individuals in a single tube) could be conducted to save time and resources. In the case that individual genotyping is necessary, these costs will be increased, particularly for 1014 positions, where variants are more difficult to distinguish from pooled samples. Hence, we estimated that the implementation of mHRM implies a 3 h for the processing of around 88 samples (one complete plate). The experimental steps include DNA purification and mHRM reactions, whereas PASA + REA involve DNA purification, five PCRs, one enzymatic digestion, and gel electrophoresis. The main advantage of PASA + REA is that it can be implemented in a moderately equipped lab (Table 3).

As expected, the results showed that L925I^kdr^ is more easily distinguished from the wild-type than L1014F by the mHRM technique. This is due to the higher shift in MT in the substitution C to A (position 925) compared with A to T (position 1014); we found that a change in MC shape is useful for complementing the genotyping in both positions. In further developments, algorithms could be designed for automatic interpretation of mHRM results by considering both MT and MC parameters [29]. In ambiguous cases or populations with mixed genotypes, mHRM could be complemented with alternative techniques, such as Sanger sequencing, PASA, and REA [9,10]. The increase in the throughput of the method with respect to previous alternatives could help in the implementation of genotype monitoring as a routine activity before insecticide treatments. In the regions with vector presence, triatomine control is a task organized and implemented by governments. This could facilitate the centralization of the genotyping activity in a single laboratory receiving samples from a wide region. Hence, the initial investment in equipment required for high-throughput genotyping methods would be justified by the reduction in time and costs.

Resistance to pyrethroids in *T. infestans* has expanded in recent years. From the original reports in the Bolivian–Argentinean border and the Chaco province in Argentina [6,30], other regions with variable resistance levels were reported [21,22]. However, the higher percentages of highly resistant populations were reported in Güemes department, Chaco province [24,25]. We did not find toxicological resistance or kdr substitutions in the previously unreported populations from Catamarca, Córdoba, Formosa, La Rioja, and Chaco. Interestingly, we found that either L925I^kdr^ or L1014F^kdr^ was consistently associated with a phenotype of high resistance to pyrethroids. We confirmed that L925I^kdr^ is characteristic of resistant populations originating from Güemes Department in Chaco Province (Argentina) and L1014F^kdr^ in those from Tarija Department (Bolivia), reinforcing the hypothesis of two resistant origins [11]. Genotyping of individuals from susceptible, middle, and highly resistant natural populations would be important to obtain further insights on the situation of resistance in the field and in the molecular background of resistance in *T. infestans*. This information will be important in both basic entomology and for a better design of vector control strategies. The early detection of populations with the potential to develop high resistance is required in order to make rational decisions in time. In this context, we present here a high-throughput tool that can accelerate the genotyping of kdr mutations associated with high resistance in the species. The implementation of safe alternatives is becoming urgent in order to complement or replace pyrethroids when resistance is detected.

## Figures and Tables

**Table 1:** List of primers used for this study.

Primer Name	5′-3′ Sequence	Melting Temperature (°C)	Amplicon Size
925 F	TGGCCAACATTGAATTTATTGATATC	62	75 bp
925 R	TAAGACAAATGTCAAATTGCCAA	62	
1014 F	CCGGCGGCGGTGTGGGACTGTATGCACGTT	62	95 bp
1014 R	CCGGCGGCGGAATATATAAAGTACTTACAAC	62	

**Table 2:** Genotypes in populations from different regions in Argentina.

Country	Province/Department	Locality	Toxicological Phenotype	VGSC Genotype Determined by mHRM
Argentina	Catamarca/Capayán	Balde de Punta	Susceptible	S
	La Rioja/San Martín	San Martín	Susceptible	S
	Córdoba/Cruz del Eje	Minas	Susceptible	S
	Formosa/Bermejo	Chiriguanos	Susceptible	S
	Chaco/Güemes	El Asustado	High resistance (a)	L925I kdr
	Chaco/Güemes	Nueva Pompeya	Susceptible	S
	Chaco/Güemes	Pampa Argentina	High resistance (b)	L925I kdr
	Chaco/Güemes	La Gerónima	Low resistance (c)	S
	Chaco/Güemes	El Juramento	High resistance (c)	L925I kdr
	Chaco/Güemes	El Malá	High resistance (d)	L925I kdr
	Chaco/Quitilipi	25 de Mayo	Susceptible	S
Bolivia	Gran Chaco/Tarija	Tierras Nuevas	High resistance (e)	L1014F kdr
	Gran Chaco/Tarija	Villa del Carmen	High resistance (e)	L1014F kdr

Toxicological phenotype described in a. Fronza et al., 2024 [25]; b. Remón et al., 2020 [26]; c. Sierra et al., 2016 [11]; d. Carvajal et al., 2012; and [27] e. Germano et. al., 2010 [16].

**Table 3:** Comparison of PASA + REA and mHRM assays for the genotyping of KDR mutations in *T. infestans*.

	HRM	REA + PASA
Equipment required (cost in USD)	Calibrated real-time thermocycler (25,000)	Thermocycler + electrophoresis + image acquisition (15,000)
Required analysis time	3 h	13 h
Workflow	Closed-tube assay	Nested PCR amplifications, restriction enzyme digestion and gel electrophoresis
Number of experimental steps	Two	Six
Primer sets required	Two primer pairs	Four primer pairs
Reaction wells/tubes required for 440 samples	96	360
Estimated reagent cost for 440 pooled samples (USD)	64	77

## Data Availability

All data supporting the findings of this study are provided in the manuscript.
